# Approaches to Establishing Tolerance in Immune Mediated Diseases

**DOI:** 10.3389/fimmu.2021.744804

**Published:** 2021-09-20

**Authors:** Michelle F. Huffaker, Srinath Sanda, Sindhu Chandran, Sharon A. Chung, E. William St. Clair, Gerald T. Nepom, Dawn E. Smilek

**Affiliations:** ^1^Immune Tolerance Network, University of California San Francisco, San Francisco, CA, United States; ^2^Immune Tolerance Network, Duke University, Durham, NC, United States; ^3^Immune Tolerance Network, Benaroya Research Institute, Seattle, WA, United States

**Keywords:** immune tolerance, allergy, transplantation, autoimmunity, clinical trial, immunomodulation, immunosuppression

## Abstract

The development of rational approaches to restore immune tolerance requires an iterative approach that builds on past success and utilizes new mechanistic insights into immune-mediated pathologies. This article will review concepts that have evolved from the clinical trial experience of the Immune Tolerance Network, with an emphasis on lessons learned from the innovative mechanistic studies conducted for these trials and new strategies under development for induction of tolerance.

## Introduction

The mission of the Immune Tolerance Network (ITN) is to advance the development of immune tolerance strategies in autoimmunity, allergy, and transplantation by conducting high-quality clinical trials with emerging therapeutic agents. Integrated mechanism-based research is a critical component of these trials that provide new insights into the success or failure of the intervention, as well as further understanding of disease pathogenesis. These results in turn provide the building blocks for further clinical trials and the design of new treatment strategies and incremental advancement towards the tolerance goal.

**Figure 1:**
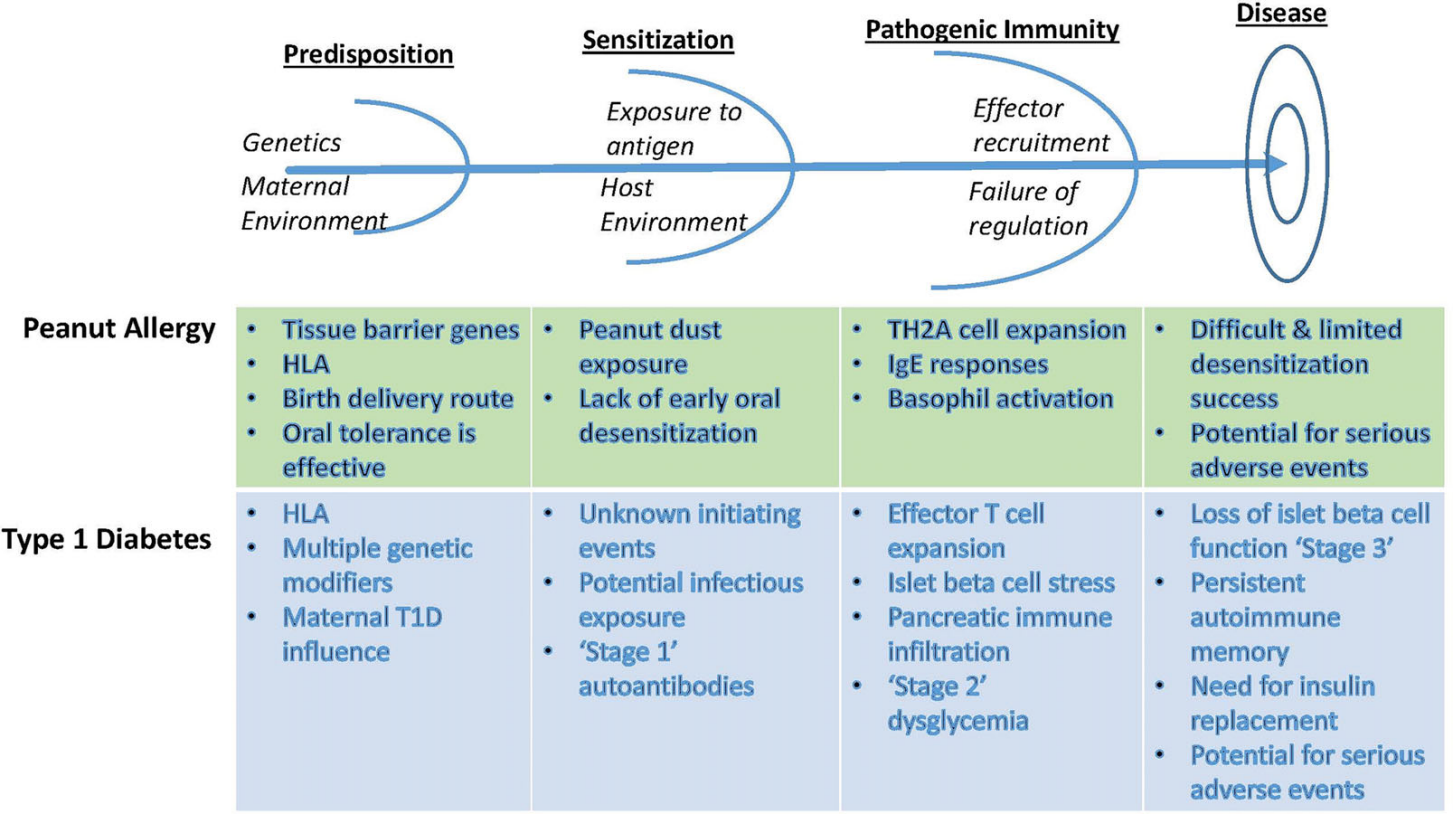
Conceptual parallels between the approach to autoimmunity and allergy tolerance trials, using examples from T1D and from peanut allergy. Identification of early at-risk children allows for prevention strategies that rely on antigen exposure in the context of immune deviation or anergy. After initial antigen sensitization, however, additional measures are required to blunt effector responses to inhibit immune amplification events. Failing this, determinant spreading elicits robust immunity that recruits additional effector pathways and conditions inflammatory innate tissue responses, now requiring combinations of targeted therapeutics to ‘reset’ the immunological threshold and enable an opportunity to reestablish homeostatic balance between regulatory and effector pathways. Recognition of these differences requires appropriate staging and monitoring in order to select therapeutic options with tolerogenic potential.

Central to the ITN’s mission is the concept of immunologic tolerance. While specific clinical definitions of tolerance vary across the immune mediated diseases, they all center on differentiating permanent or prolonged improvements in disease that represent a significant clinical benefit over the expected natural history of disease or the standard of care. Conceptual parallels are illustrated in **Figure 1**. In the transplant setting, tolerance is defined as graft acceptance without the need for a continuous immunosuppressive regimen, or a greatly reduced regimen. In allergic disease, tolerance may be defined as prolonged unresponsiveness to antigen challenge or exposure after withdrawal of allergen immunotherapy. For autoimmune diseases, tolerance is reflected in reduced need for disease-modifying therapy or prolonged improvement of disease manifestations, such as retention of residual insulin secretion in type 1 diabetes.

However, at a molecular level, immunologic tolerance represents prolonged or permanent modulation of aberrant immune responses towards a homeostatic state. Immune self-tolerance normally occurs in T and B lymphocytes by central and peripheral mechanisms, reviewed previously (1). Central tolerance involves elimination of lymphocytes with high affinity receptors for self-components, a process that takes place in T lymphocytes and B lymphocytes during cellular maturation in the thymus and bone marrow, respectively. Self-reactive lymphocytes that are not eliminated centrally during development exit into the periphery where they are restrained by a variety of tolerance mechanisms, included induction of anergy, cellular exhaustion, and suppression by regulatory T cells, B cells, innate immune cells, and inhibitory cytokines.

In disease conditions, immunologic tolerance can in theory be re-established through a variety of mechanisms which may expand or augment regulatory cells (e.g. Tr1, Treg, Breg) and suppress effector responses (e.g. effector cell depletion, co-stimulation blockade, anti-cytokine therapy) that in tandem act to restore immune homeostasis and disease quiescence. In this review, we provide an overview of ITN successes, challenges, and new strategies to achieve immune tolerance in the fields of allergy, autoimmune disease, and solid organ transplantation. **Table 1** summarizes the ITN trials mentioned in this review. Summaries of all ITN trials are available at www.immunetolerance.org.

**Table 1:** Summary of ITN Clinical Trials Included in this Review.

Disease Indication	Trial Name	Intervention	Status	References	ClinicalTrials.gov Identifier
**Allergy**					
At risk for peanut allergy	LEAP LEAP-On	Peanut	Complete	(2–7)	NCT00329784 NCT01366846
Peanut allergic	IMPACT	Peanut	Complete	(8)	NCT01867671
At risk for atopy	ACTIVATE	Vaginal microbiome seeding	Ongoing		NCT03567707
Grass allergic	GRADUATE	Timothy grass + Dupilumab	Ongoing		NCT04502966
Grass allergic	GRASS	Timothy grass	Complete	(9)	NCT01335139
Cat allergic	CATNIP	Cat allergen + Tezepelumab	Complete		NCT02237196
					
**Transplantation**					
Kidney transplant	Mixed Chimerism	Immunosuppression withdrawal following donor hematopoietic stem cell transplant	Complete	(10)	NCT00063817, NCT00801632
Kidney transplant	TEACH	Immunosuppression withdrawal following donor mesenchymal stromal cells	Ongoing		NCT03504241
Liver transplant	LITTMUS	Immunosuppression withdrawal following donor specific Tregs	Ongoing		NCT03577431, NCT03654040
HLA sensitization awaiting kidney transplant	ADAPT	Carfilzomib plus belatacept	Planned		NCT05017545
	ATTAIN	Daratumumab plus belatacept	Planned		NCT04827979
					
**Autoimmunity**					
Treatment-resistant multiple sclerosis	HALT-MS	Autologous hematopoietic stem cell transplant Autologous hematopoietic stem cell transplant	Complete	(11–13)	NCT00288626
	BEAT-MS		Ongoing		NCT04047628
ANCA associated vasculitis	RAVE	Rituximab	Complete	(14)	NCT00104299
Lupus nephritis	CALIBRATE	Rituximab + Belimumab Rituximab + Belimumab	Complete	(15)	NCT02260934
Primary membranous nephropathy	REBOOT		Ongoing		NCT03949855
Antiphospholipid syndrome	DARE-APS	Daratumumab	Planned		Pending
New onset type 1 diabetes New onset type 1 diabetes New onset type 1 diabetes	AbATE	Teplizumab	Complete	(16–18)	NCT00129259
	T1DAL	Alefacept	Complete	(19–21)	NCT00965458
	START	Antithymocyte globulin	Complete	(22)	NCT00515099
Lupus nephritis	ACCESS	Abatacept Abatacept	Complete	(23)	NCT00774852
Multiple sclerosis	ACCLAIM		Complete	(24, 25)	NCT01116427
Psoriasis vulgaris	PAUSE	Ustekinumab + Abatacept	Complete	(26)	NCT01999868

Summaries of all ITN trials are available at www.immunetolerance.org.

## Barriers to Tolerance

Immunological memory is a hallmark of successful immune responses, and is essential for pathogen surveillance and extinction. It is also a formidable barrier to successful immune tolerance induction. The challenge of reversing pathogenic immune responses in individuals with autoimmune disease and allergy requires not only directed therapy against immune effector cells, but also prevention of recurrent memory responses when therapy is discontinued. This concept also plays a role in transplantation, both through initial heterologous memory and subsequently in dealing with the robust alloimmune response. Innate immune activation, memory, and self-perpetuating inflammatory cascades must be restrained, allowing tissue repair to occur. Therefore, a major focus of immune tolerance strategies is to retain and expand regulatory immune mechanisms, thereby exploiting homeostatic pathways that are intrinsic to a healthy balance of immune effector and regulatory compartments.

**Figure 2:**
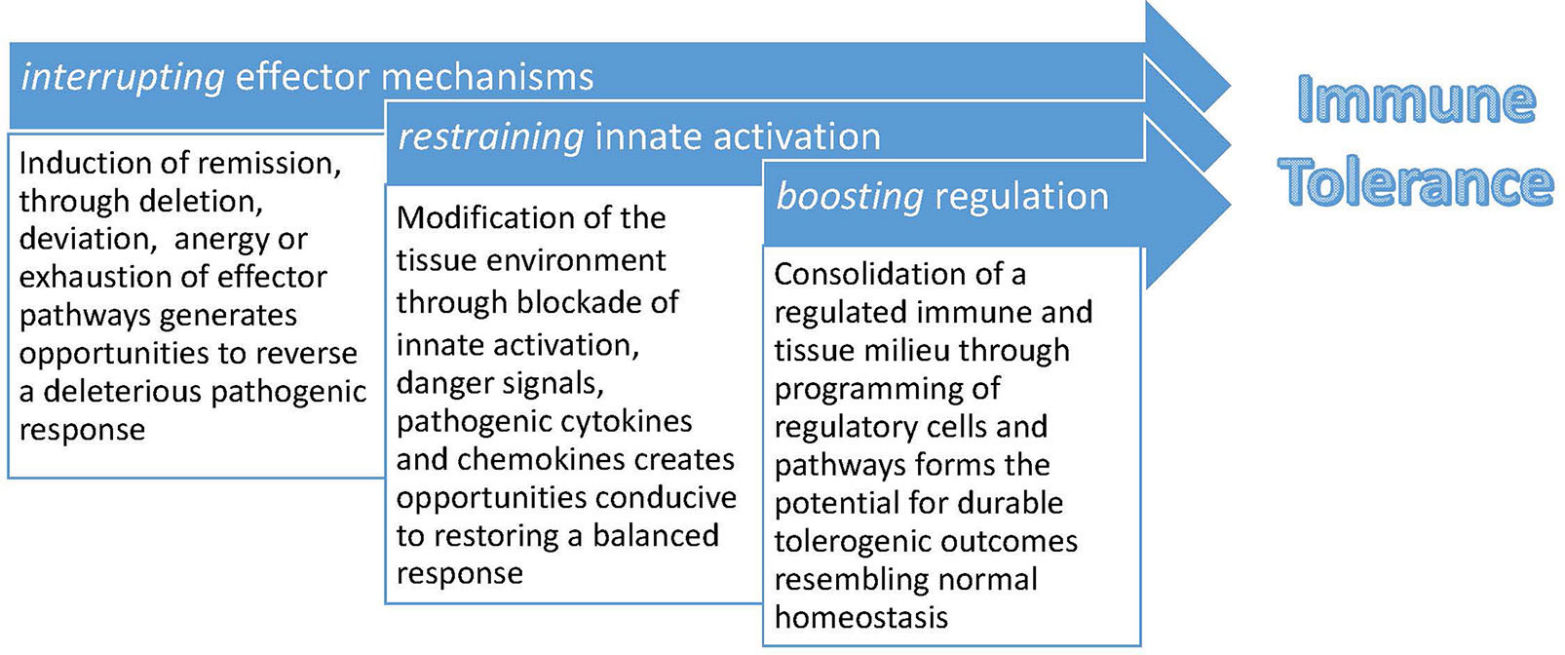
A conceptual framework for induction and maintenance of immune tolerance. Tolerogenic therapies that focus on boosting regulatory immune responses face daunting hurdles in the form of established immunological memory and robust redundant effector activation pathways, involving both adaptive and innate immunity. Creating a host environment conducive to regulation is augmented by first inducing clinical remission and decreasing tissue inflammation. This concept creates a practical platform for combination therapies in which short-term immunosuppressive or immunomodulatory interventions are first employed, followed by emphasis on restoration of immune regulation.

This combination of *interrupting* effector mechanisms, *restraining* innate activation, and *boosting* regulation is the central dogma of successful immune tolerance therapy, illustrated in **Figure 2**. Therapies that achieve only one of these goals without the others, as discussed in the examples below, achieve suboptimal or transient clinical benefit. And because immunological memory is very resilient, early intervention – at the time of transplant or early in the immune process prior to the onset of clinical disease in allergy or autoimmunity – can be attempted whenever at-risk individuals can be identified.

Underlying these efforts to rebalance immune effector and regulatory responses is the recognition that drastic immune suppression is accompanied by significant safety concerns, notably infectious and neoplastic risk, as well as life-long drug administration. Consequently, the targets of immune tolerance therapy need to be selective, addressing the ITN’s ‘*interrupting/restraining/boosting’* therapeutic dogma without creating an unacceptable level of danger. Selective targets within the adaptive immune system may involve specific subsets of T or B cells, or even antigen-specific receptors and signaling pathways; and selectivity in a broader sense may target a limited set of cytokine, chemokine, or activation pathways without global immunosuppression. Rapid development over the last two decades of novel immune therapeutic agents that meet these types of selectivity requirements has enabled the design of clinical trials with aspirations for achieving immune tolerance. Although transplantation, allergy, and autoimmune diseases have many distinct characteristics, conducting such trials within the ITN provides an opportunity to study immune mechanisms and concepts across clinical disciplines while evaluating safety, therapeutic durability, and homeostatic immune reconstitution.

## Allergy

Desensitization strategies have been a mainstay of allergy therapy for many years, for many types of environmental allergens, including hymenoptera venom and aeroallergens such as pollens, dust mites, and animal dander. On this background of clinical experience showing that allergen specificity of an allergic response that can often be modulated by controlled antigen exposure, the ITN has designed several clinical trials intended to achieve durable, long-lasting allergen desensitization, i.e. tolerance.

IgE-mediated peanut allergy typically develops in the first two years of life and for the majority affected, the disease persists into adulthood. Sensitization to peanut may involve cutaneous and enteral pathways that lead to production of TH2 cytokines and antigen-specific IgE. The Learning Early about Peanut Allergy (LEAP) trial demonstrated that early oral exposure to peanut prevented the development of peanut allergy in atopic infants including those who had already developed sensitization to peanut (2). This protection proved to be durable through the LEAP-On trial, which assessed peanut allergy status after one year of avoidance of peanut following completion of the oral peanut intervention (3). Nearly complete protection in the LEAP cohort of infants with family histories of peanut allergy and eczema, who were at high risk for development of peanut allergy, led to the publication of new public health guidelines for safely introducing peanut protein into the early childhood diet (4). This protection was highly allergen specific, as consumption of peanut did not prevent the development of other food allergies (5).

While it is possible that antigen specific prevention may be applicable for many food allergens, there are practical limitations to introducing multiple allergens in young infants, including the ability of young infants to comply with ingestion of multiple different foods in sufficient quantity to prevent allergic disease (27). Prevention strategies are needed that are personalized based on individual risk of developing a specific allergy. For example, subsequent whole genome sequencing from the LEAP trial revealed a novel association for peanut allergy with a single nucleotide variant in the mucosa-associated lymphoid tissue lymphoma translocation (*MALT1*) gene (6). *MALT1* encodes a paracaspase that acts in response to antigen binding to the T-cell or B-cell receptor leading to NFκB activation (28). The association of *MALT1* with peanut allergy was found to be independent of atopic dermatitis and egg allergy, suggesting that carrier status predisposed to a unique risk for peanut allergy specifically, and correlated with the progressive acquisition of IgE antibodies to multiple allergenic peanut protein components (6). This immunological relationship was further explored by analyzing the development of peanut specific IgE to a broad repertoire of linear epitopes after the second year of life (7). Peanut specific IgE in infants sensitized to peanut and consuming it, however, recognized conformational epitopes without expansion of peanut specific IgE reactivity to linear epitopes. These findings suggest an interaction between genetics, age of peanut exposure, and likelihood of tolerance, opening possibilities for personalized prevention and intervention strategies based on distinct phenotypic and genotypic risk factors.

Observations in another ITN study, the Oral Immunotherapy for Induction of Tolerance and Desensitization in Peanut-Allergic Children (IMPACT) trial, further emphasize this point. IMPACT was a randomized double-blind placebo controlled trial of peanut oral immunotherapy (OIT) in peanut allergic children ages 12-48 months. Successful desensitization at the end of OIT and persistent desensitization after 6 months of withholding OIT was associated with lower baseline peanut specific IgE, particularly in children below the age of 3 years (8). While tolerance was achieved in ~70% of the younger children with low initial IgE, overall the success rate was much lower, indicating an opportunity may exist early in disease during which the host immune response may be more receptive to development of tolerance. Together, LEAP and IMPACT demonstrate that the atopic march, the paradigm for the progression from atopic dermatitis to allergen-specific disease, can be halted or even reversed early in its course for at least a single allergen-specific disease.

These studies illustrate the concept that the younger immune system, not surprisingly, is more amenable to tolerization using antigen desensitization. They also suggest opportunities to attempt tolerization therapy in older, more established allergy, using agents to encourage an immune response that resembles the immature phenotype. Towards that end, two novel strategies are currently under ITN development, one using microbiome immune modulation and another using anti-cytokine agents in combination with desensitization. Microbiome modification is conceptually a way of providing an innate adjuvant to encourage tolerization, and potentially provide a complementary approach to antigen-specific preventative therapy by inhibiting the development of atopic diseases. Differences in environmental exposure and associated alterations of the microbiome (e.g., gut, nasopharyngeal, airway epithelium) have been implicated in the development of asthma and allergic disease (29). In the LEAP trial, colonization with *S. aureus* amongst young participants was associated with increased IgE production, persistence of egg allergy, and inhibition of oral tolerance to peanut (30). In the Copenhagen Prospective Studies on Asthma in Childhood 2010 mother-child cohort, the risk of asthma at 6 years [OR 2.45 (95% CI 1.32 to 4.55), P = 0.004] and allergic sensitization at 18 months [OR 1.68 (95% CI 1.01 to 2.79), P = 0.046] was higher in infants delivered by Cesarean section compared to vaginally-delivered infants. Since the increased risk of asthma was identified in infants born by Cesarean section whose gut microbiome was less mature at 1 year of age (i.e., retained a Cesarean section microbiome signature), it is possible the infant gut microbiome may be a contributing factor to this predisposition for the development of asthma (31). In addition, in neonates who have a high risk for developing asthma, the gut microbiome produces metabolites, which in murine studies have been shown to increase pulmonary inflammation and decrease regulatory T (Treg) cell abundance in the lung (32). Taken together, these studies suggest that altering the newborn microbiome could favorably impact the risk of allergic and atopic disease. To address this hypothesis, the Vaginal Microbiome Exposure and Immune Responses in C-section Infants (ACTIVATE) trial is studying the impact of vaginal microbiome seeding on the development of allergic disease in infants delivered by Cesarean section (NCT03567707). Longitudinal samples of the gut, skin, nasal, and oral microbiomes will be collected over the child’s first 3 years of life to assess for compositional factors as well as changes to the microbiome (e.g., maturation) associated with developing sensitization to food and aeroallergens.

In older individuals, with established allergic responses and mature immunological memory, a strategy to alter the allergenic immune program associated with TH2 T cell immunobiology is an alternative approach. To this end, the ITN GRADUATE trial, which is currently underway (NCT04502966), combines dupilumab (anti-IL4R) and sublingual grass desensitization to induce durable tolerance. This study builds on the previous ITN GRASS trial comparing desensitization to grass pollen between the sublingual and subcutaneous routes (9). Another cytokine strategy, using tezepelumab (anti-TSLP) to alter TH2 developmental programming, has been studied recently in the ITN CATNIP trial as a potential adjunct to desensitization in established allergic patients (NCT02237196). With recent advances in understanding TH2 diathesis, agents such as anti-OX40 (33), or other strategies targeting allergenic T effector cell subsets, known as TH2A cells (34), may provide additional therapeutic opportunities.

## Transplantation

Hematopoietic chimerism has been utilized to induce tolerance of kidney allografts in animals and humans, based on the foundational discovery that dizygotic cattle twins exhibit stable red cell chimerism and mutually accept skin grafts (35, 36). A proof of concept for chimerism as a robust mechanism of transplant tolerance was provided by recipients of allogeneic hematopoietic stem cell transplant for leukemia who did not require maintenance immunosuppression after kidney transplant from the same donor 3–11 years later (37). The ITN clinical experience, while successful at achieving transient mixed chimerism (2-3 weeks) in haplotype-matched patients, was only able to induce durable immunosuppression free graft survival in a small number of study participants (10). Similar loss of chimerism and subsequent rejection has been seen by others (38, 39), likely indicating a need for improved peripheral deletion of donor-reactive T cell clones and induction of regulatory mechanisms. Use of a donor hematopoietic stem cell product featuring CD8+/TCR- “tolerance-promoting facilitating cells” (40), various regulatory T cell strategies (41–44), or a donor mesenchymal stem cell infusion (the ITN TEACH trial, NCT03504241) are alternatives currently being evaluated for potential for inducing transplant tolerance using therapeutic cell transfer. The ITN LITTMUS trials (NCT03577431, NCT03654040) are currently testing whether infusion of alloantigen‐specific regulatory T cells, generated from liver transplant recipient cells collected within the first post-transplant year, can facilitate withdrawal of immunosuppressive anti-rejection medications.

In transplantation, the importance of controlling immunological memory cannot be overemphasized. A significant proportion of transplant candidates have accumulated pre-formed antibodies to HLA antigens as a result of prior sensitizing events such as blood transfusion, pregnancy, or a previous transplant. These patients represent a growing challenge for the transplant community as their sensitized immune system makes it more difficult to find compatible potential donors. The achievement of “HLA desensitization” may be therefore considered a waypost on the road to B-cell tolerance. Trials of B cell depletion using rituximab and obinituzumab, although attractive in theory, were found to be unsuccessful in practice, likely reflecting the inability of these agents to eliminate long-lived plasma cells, as well as to sufficiently diminish memory B cells that are destined to develop into anti-HLA antibody-secreting cells (45, 46). Interestingly, the strategy of targeting antibody-secreting plasma cells alone, using agents highly effective against multiple myeloma, has also been found to be insufficient in producing a durable response (47). Elegant studies in allosensitized non-human primate (NHP) models have now shown that ensuing compensation by expanding germinal centers (GC) following plasma cell depletion underlies the rapid repopulation of plasma cells and consequent rebound in HLA antibody (48). Costimulation *via* the CD28 and CD40 pathways plays a critical role in GC interactions between Tfh cells and B cells (49, 50), and costimulation blockade in NHP has been shown to collapse GC and abrogate the humoral rebound seen after plasma cell depletion (51). Based on these observations, a “dual-targeting” strategy of HLA desensitization consisting of 1) plasma cell depletion (using proteasome inhibitors or anti-CD38 monoclonal antibody) and 2) suppression of the upstream humoral response by costimulation blockade is now being tested in two parallel ITN trials (ADAPT, NCT05017545, and ATTAIN, NCT04827979).

## Autoimmunity

Rational strategies for tolerance induction in autoimmune diseases rely on alignment of pathogenic mechanisms and potential therapeutic targets. In some cases, selective targeting of effector T or B cell compartments may be an attractive way to induce remission, by creating a tissue environment less resistant to subsequent homeostatic regulation; in other cases, heterogeneity among patients with similar diseases may require more personalized strategies to optimize the likelihood of matching the appropriate therapy with an individual subject. Numerous ITN trials, in diseases such as multiple sclerosis, systemic lupus erythematosus (SLE), anti-neutrophil cytoplasmic antibody (ANCA)-associated vasculitis, type 1 diabetes, and psoriasis, have documented robust changes in immune cell compartments in association with T or B cell targeting, but clinical responses are often transient. Therefore, different ITN trials have been designed to evaluate agents for selective immune depletion, immune modulation, and immune regulation, with combinations of those agents when feasible to assess synergistic mechanisms of action. A few examples that illustrate key concepts are discussed below.

Although over a dozen disease-modifying agents have been approved by the FDA for the treatment of multiple sclerosis (MS), some patients with this disease are resistant or refractory to treatment and continue to relapse and accumulate disability. In treatment-resistant relapsing MS, an immune “reset” can be accomplished with immunoablation followed by autologous hematopoietic stem cell transplant (AHSCT). In this procedure, the pathogenic immune repertoire is eliminated with interruption of autoimmune destruction of myelin in the central nervous system. Clinical trials, including the HALT-MS trial conducted by the ITN, demonstrated high efficacy in aggressive treatment-resistant relapsing MS (11, 12, 52–54). There is evidence that AHSCT alters the immune system upon reconstitution *via* thymic reactivation (55), rebalancing of regulatory and effector immune components (56–59), and T cell repertoire diversification (13). In the HALT-MS trial, dominant CD4+ T cell clones were largely replaced by a new repertoire following transplant, while CD8+ T cell clones re-emerged and better clinical outcomes post-transplant were associated with a more diverse CD8+ T cell repertoire (13). To confirm this result, the BEAT-MS multicenter trial is in progress comparing the efficacy and cost-effectiveness of AHSCT to high efficacy disease modifying agents in treatment-resistant relapsing MS (NCT04047628), with the aim of developing high quality evidence supporting consensus recommendations for utilizing AHSCT to treat resistant forms of relapsing MS (60).

Depletion of specific effector cell populations without the global changes induced by AHSCT are also effective, although with inconsistency in the durability of response. In ANCA-associated vasculitis, ANCA do not appear to cause disease by forming immune complexes or directly binding to tissues. Instead, these autoantibodies are suspected to bind neutrophils, resulting in hyperactivation of neutrophils and formation of neutrophil extracellular traps, which in turn results in vascular damage. The Rituximab for ANCA-associated Vasculitis (RAVE) trial treated patients with severe active granulomatosis with polyangiitis and microscopic polyangiitis with rituximab (anti-CD20), with the goal of depleting antibody-secreting B cells and promoting sustained disease control (14). In this study, rituximab therapy was shown to be non-inferior to cyclophosphamide, the previous standard of care for induction of disease remission. Following induction of clinical remission with rituximab therapy, the majority of patients nevertheless relapsed, indicating that immune tolerance was not achieved despite the initial clinical response. A similarly transient benefit of rituximab therapy was seen in the TrialNet study of type 1 diabetes (61, 62); interestingly, T cell transcripts indicating activation following B cell depletion correlated with reduced preservation of residual insulin secretion, suggesting a need for targeting multiple effector arms of the immune response (63).

Despite an abundance of evidence supporting a critical role for B cells in the pathogenesis of SLE, rituximab-induced B cell depletion has failed in randomized, controlled trials to show clinical efficacy in both non-renal SLE and lupus nephritis (64, 65). One possible explanation for the early failure of rituximab therapy for SLE in clinical trials may be that levels of B-cell activating factor (BAFF) increase following B cell depletion (66). In BAFF-transgenic mice, elevated levels of BAFF rescue autoreactive B-cells and prevent them from becoming anergic (67). To address the hypothesis that inhibition of BAFF following B cell depletion might improve disease control in this setting, the ITN CALIBRATE trial was undertaken to explore in lupus nephritis the efficacy of adding the anti-BAFF agent belimumab to a B cell depletion regimen combining rituximab and cyclophosphamide. Although the percentage of autoreactive naïve B cells were decreased in the group who received belimumab, the addition of a BAFF blocker did not differentiate the clinical outcomes in lupus nephritis (15). A related rituximab and belimumab combination strategy is also being studied in primary membranous nephropathy, where anti-PLA2R autoantibody production is being assessed in the ITN REBOOT trial. In this disease, in contrast to SLE, rituximab therapy has clinical efficacy for some patients. Belimumab will be initiated 4 weeks prior to rituximab therapy based on the observation that circulating memory B cells (CD19+/CD20+/CD27+) increase in numbers after belimumab therapy (68). Thus, initiating belimumab therapy prior to the intervention with rituximab use has been hypothesized to increase the proportion of memory B cells that can be depleted by this CD20-depleting antibody.

Similar to the rationale for transplantation studies noted above, plasma cell targeting is an attractive option for some antibody-mediated autoimmune diseases. Elimination of plasma cells could be beneficial for the treatment of antibody mediated diseases such as antiphospholipid syndrome (APS), a systemic autoimmune disease characterized by thrombotic and obstetric manifestations in individuals with potentially pathogenic antiphospholipid antibodies (69). Standard treatment of APS is lifelong anticoagulation, which is not always effective. Patients with APS often have persistence of antiphospholipid antibodies and are therefore at risk for future thrombosis, shining light on the antibody-secreting cell as a plausible target for effective therapy. A candidate drug for targeting the antibody-secreting plasma cells in APS and other autoimmune conditions is daratumumab, a cytolytic monoclonal antibody developed for the treatment of multiple myeloma. Daratumumab binds to CD38 expressed on plasma cells and is cytolytic for these cells. Small case studies of patients with APS, SLE, and autoimmune hematologic conditions report that treatment with daratumumab reduces autoantibody production in concert with improved clinical manifestations of disease (70–72), providing the rationale for the ITN study of daratumumab therapy in APS with the goal of eliminating antiphospholipid antibodies.

In parallel with these depletional studies that specifically target the pathologic humoral response, other ITN trials have focused on inducing remission and tolerance in autoimmunity through T cell depletion and modulation. The ITN trials of teplizumab (anti-CD3), alefacept (LFA3-Ig), and anti-thymocyte globulin (ATG) in type 1 diabetes (T1D) are particularly informative. Teplizumab is a monoclonal anti-CD3 antibody, investigated by the ITN for over a decade in several clinical trials. In the ITN AbATE study, treatment with teplizumab delayed the loss of residual insulin secretion in patients with type 1 diabetes in a subset of subjects, with some maintaining insulin secretory function for several years (16). Teplizumab does not deplete T cells; rather, it acts as a partial agonist to induce a series of functional and phenotypic changes. For example, whole blood transcriptomic analysis in AbATE identified *EOMES*, a transcription factor and marker of T-cell exhaustion, as a correlate of changes in insulin secretion (16–18). Moreover, CD8 effector memory T cells were found to have greater expression of EOMES mRNA and higher expression levels of other exhaustion markers, namely KLRG1 and TIGIT, by flow cytometry. These KLRG1+ TIGIT+ CD8 T-cells showed impaired expression of cell cycle genes when activated, suggesting impaired proliferative capacity, and an effector T-cell exhaustion profile that correlated with improved outcomes. These studies indicate a novel mechanistic basis for tolerance, namely induction of lymphocyte exhaustion by a T cell agonist therapy. The TrialNet TN10 trial subsequently extended these findings to a population of high-risk individuals who had anti-islet cell antibodies, a marker of susceptibility to T1D, but who were not yet hyperglycemic. In this study, teplizumab induced a similar T cell exhaustion profile and resulted in an overall delay in the development of T1D by a median of 24 months (73).

The costimulatory CD2 cell surface molecule is most prominently expressed on CD4 T effector memory (Tem) cells and naïve CD8 (19). CD4 T regulatory cells (Treg) express CD2 at lower levels than any other T-cell subset. Targeting this pathway to affect the antigen-specific effector memory response preferentially over the regulatory T-cell compartment was the basis for the ITN T1DAL trial. T1DAL was a clinical trial carried out in children and adolescents with new-onset type 1 diabetes and investigated the clinical efficacy of alefacept, an LFA-3 fusion protein that blocks the costimulatory LFA-3/CD2 interaction (19–21). In this study, 30% of treated subjects retained or improved their insulin secretory function over 2 years, and another 40% showed only modest loss of islet function. Similar to treatment with teplizumab, alefacept treatment was associated with increases in the prevalence of CD8 effector memory cells expressing both KLRG1 and TIGIT; as seen in AbATE, these exhausted CD8 cells correlated with beneficial clinical response. Notably, however, in T1DAL they appeared in the blood approximately 9 months after therapy, following an earlier induction of PD1 on a CD4 Tem population. Both teplizumab and alefacept treated participants demonstrated these changes in effector populations concurrent with relative preservation of regulatory T cells, providing a proof-of-concept for the sequential combination of an induction therapy followed by consolidation of the regulatory response. This conclusion was reinforced by the findings in the ITN START trial (22) which utilized anti-thymocyte globulin in a similar study of patients with early T1D. In START, no therapeutic benefit was observed, and analysis of lymphocyte subsets indicated depletion of multiple cell lineages, notably including both regulatory and effector T cell populations. Retention or boosting of regulatory responses is therefore a major objective of current ITN therapeutic strategies, forming one of the key components in clinical trial designs.

Failure to retain regulatory T cell function may explain the disappointing outcomes in several trials using CD28 costimulatory blockade as a therapeutic tolerance strategy. Abatacept (CTLA4-Ig) is a fusion protein consisting of the extracellular domain of the CTLA4 ligand for CD80/86 coupled to a modified Fc portion of human immunoglobulin G (IgG). It acts by preventing CD80/CD86 on APCs from binding to CD28 on T cells, thereby inhibiting T cell activation and function, and serving as an effective treatment option in adult rheumatoid arthritis and juvenile idiopathic arthritis. ITN trials with abatacept in multiple sclerosis, lupus nephritis, and psoriasis, however, did not demonstrate clinical benefit for induction or maintenance of tolerance (23, 24, 26). Analysis of peripheral blood cells from the participants in the multiple sclerosis trial demonstrated that the relative proportions of activated CD4+ T follicular helper cells and regulatory T cells were both decreased in participants receiving abatacept compared with those receiving placebo (25). Similar changes following abatacept treatment, including loss of regulatory T cells, have been observed by others in a variety of other diseases (74–76). While transient immunomodulatory effects in effector cells from CD28 blockade occur, these require continued drug administration, and therefore the undesirable inhibition of regulatory T cells that also utilize CD28-dependent pathways appears to preclude effective use of abatacept as a tolerance therapy where drug discontinuation is desired.

## Discussion

Successful treatment interventions in transplantation, allergy, and autoimmune disease rarely allow for discontinuation of therapy and result in sustained and selective immune tolerance. However, they do occur, and in the context of clinical trials, they provide an opportunity to identify specific therapeutic targets, or combinations of targets, capable of restoring homeostatic immunity. Notably, minimization or extended drug holidays from immunosuppressive therapy also provide clinical benefit, even without achieving the ideal goal of permanent immune tolerance. Ongoing maintenance intervention may be required, for example a small dose of oral peanut (or other allergen) to maintain a non-allergic state in an individual who had previously undergone successful desensitization to that allergen.

Looking across multiple clinical trials and different clinical disciplines in ITN studies, shared patterns emerge that emphasize the need for a combination of *interrupting* effector mechanisms, *restraining* innate activation, and *boosting* regulation. These objectives can be met through targeting of different cells and pathways in different diseases, whether B cell, plasma cell, T effector, T regulatory, cytokine or other non-lymphocytic effector mechanisms. Early intervention, and mechanism-based stratification of individuals for optimal targeting of particular immune pathways, are likely to improve success rates. Combining antigen exposure in allergic disease and transplantation with selective immunomodulation, and using initial induction strategies in autoimmunity followed by regulatory enhancement, are promising conduits for improving clinical outcomes. In the examples summarized in this article, we have learned that early intervention in the form of antigen introduction can be successful in preventing and treating peanut allergy. In autoimmune disease, immunomodulatory agents targeting T cells and inducing exhaustion pathways can delay clinical disease onset and improve the effector/regulatory T cell balance in T1D, while targeting B cells improves outcomes in ANCA-associated vasculitis. For other autoimmune diseases resistant to B cell depletion or costimulatory blockade, targeting alternative costimulatory pathways and antibody-producing plasma cells in tandem, for example, and combination strategies interrupting key hubs in the adaptive and innate immune response may be required to restore immune tolerance and tissue homeostasis. In solid organ transplantation, success has been partially achieved with an immune reset *via* hematopoietic chimerism, but other strategies including cell-based therapies and molecularly targeted agents are under investigation. Eliminating unwanted immune responses in allergy, autoimmunity, and transplantation and restoring a healthy balance of regulatory and effector immune elements is a formidable goal. Nevertheless, improved understanding of the complexities of the immune system provide opportunities that continue to provide a foundation for future tolerance-inducing strategies and success.

## Author Contributions

All authors contributed to the article and approved the submitted version.

## Funding

The work of the authors was supported by the National Institute of Allergy and Infectious Diseases of the National Institutes of Health under Award Number UM1AI109565.

## Author Disclaimer

The content is solely the responsibility of the authors and does not necessarily represent the official views of the National Institutes of Health.

## Conflict of Interest

The authors declare that the research was conducted in the absence of any commercial or financial relationships that could be construed as a potential conflict of interest.

## Publisher’s Note

All claims expressed in this article are solely those of the authors and do not necessarily represent those of their affiliated organizations, or those of the publisher, the editors and the reviewers. Any product that may be evaluated in this article, or claim that may be made by its manufacturer, is not guaranteed or endorsed by the publisher.
